# Supraventricular Tachycardia Ablation in the Elderly—Characteristics and Outcomes

**DOI:** 10.1002/joa3.70283

**Published:** 2026-02-01

**Authors:** Yi Yi Chua, Julian Cheong Kiat Tay, Eric Tien Siang Lim, Germaine Jie Min Loo, Wei Sheng Jonathan Ong, Xuanming Pung, Daniel Thuan Tee Chong, Kah Leng Ho, Chi Keong Ching

**Affiliations:** ^1^ Department of Cardiology National Heart Centre Singapore Singapore Singapore

**Keywords:** cardiology, catheter ablation, electrophysiology, supraventricular, tachycardia

## Abstract

**Background:**

Catheter ablation is an effective treatment for symptomatic supraventricular tachycardia (SVT). Most studies target the general adult population; data on the elderly are less robust. We studied the clinical and procedural characteristics and outcomes in elderly patients undergoing SVT ablation.

**Methods:**

All patients undergoing atrioventricular nodal re‐entry tachycardia (AVNRT), atrioventricular re‐entry tachycardia (AVRT), and/or atrial tachycardia (AT) ablation between May 2011 and May 2022 at a tertiary center were included. Cases with concurrent ablation of atrial flutter, atrial fibrillation, and ventricular arrhythmias were excluded. Clinical and procedural characteristics and outcomes were compared between patients aged ≥ 70 years and those aged < 70 years.

**Results:**

There were 1758 cases of SVT ablation; 1608 patients were < 70 years old, and 150 patients were ≥ 70 years old. Elderly patients were more likely to have underlying structural heart disease and/or ischemic heart disease, more likely to have AVNRT and less likely to have AVRT (*p* < 0.001). Consequently, elderly patients were more likely to undergo right‐sided ablation (*p* < 0.001). The use of stereotaxis, intracardiac echocardiography, and electroanatomical mapping did not differ significantly. Procedure time, radiofrequency application time and fluoroscopy time were shorter in elderly patients (*p* < 0.05). Importantly, immediate complication and success rates did not differ significantly.

**Conclusion:**

In our study, the acute success rates are high, and complication rates are low across both cohorts despite differences in clinical and procedural characteristics. SVT ablation should be considered for symptomatic patients regardless of age. Further data including patient comorbidities and longer‐term outcomes may help patient selection.

## Graphical abstract

This single‐center retrospective study found that supraventricular tachycardia (SVT) ablation is safe and effective in younger and elderly patients. It should be considered for all symptomatic individuals regardless of age. However, the type of SVT and the presence of comorbidities, including frailty, should be considered in the clinical decision‐making process.

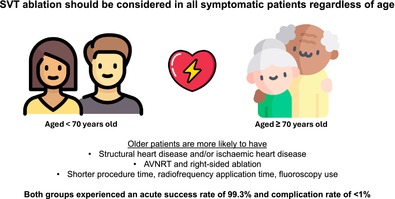

## 1 Introduction

Supraventricular tachycardia (SVT) is a dysrhythmia that is frequently encountered in clinical practice. Its estimated incidence is 36 per 100 000 persons per year, and the prevalence is 2.29 per 1000 persons [1]. SVTs are conventionally classified into atrioventricular nodal re‐entry tachycardia (AVNRT), atrioventricular re‐entry tachycardia (AVRT), and atrial tachycardia (AT). SVT affects all ages, though the predominant type of SVT differs between age groups. AVNRT and AT are more prevalent in elderly patients [2]. After the age of 65 years, the risk of developing SVT is five times greater than in young individuals [3]. This may be related to age‐related structural and electrical remodeling of cardiac tissue [4].

While SVT is typically not life‐threatening, patients are frequently symptomatic, presenting with palpitations, dizziness, dyspnoea, and syncope [5]. Elderly patients are more likely to present with syncope or presyncope, angina or heart failure, more frequently require hospitalization and emergency room treatment, and are more likely to suffer from SVT‐related mortality [4, 6, 7]. These age‐related differences in presentation and outcomes are expected given the higher prevalence of underlying cardiovascular comorbidities in elderly patients.

Radiofrequency catheter ablation (RFCA) is the first‐line therapy for symptomatic, recurrent SVT [3, 8]. It is frequently curative and often negates the need for long‐term pharmacological therapy, leading to significant improvements in patients' quality of life. With regards to long‐term management in the elderly specifically, the current American College of Cardiology (ACC) guidelines recommend that “diagnostic and therapeutic approaches to SVT should be individualised in patients more than 75 years of age to incorporate age, comorbid illness, physical and cognitive functions, patient preferences, and severity of symptoms” [8]. Most studies target the general adult population; the data on elderly patients are less robust. The aims of this study were to identify the differences in clinical and procedural characteristics and outcomes in elderly patients (age ≥ 70 years) as compared to younger patients undergoing SVT ablation.

## 2 Methods

All cases of AVNRT, AVRT, and AT ablation between May 2011 and May 2022 at a tertiary center were included. Cases with concurrent ablation of ventricular arrhythmias, atrial fibrillation (AF) and atrial flutter were excluded. Ethical review and approval were waived for this study due to use of anonymized data from another registry.

All procedures were performed under intravenous sedation with midazolam and fentanyl as required. Local anesthetic with lignocaine was administered at the femoral vascular access sites and vascular access was acquired under ultrasound guidance in all patients. When access to the left side of the heart was required, this was achieved using either the retrograde aortic approach or transeptal approach with or without intra‐cardiac echocardiography (ICE) guidance, according to the operator's preference. Fluoroscopy was performed using Philips FD10 (Philips Electronics North America, Andover, MA, USA). The minimum dose compatible with adequate imaging was used during the advancement of the catheters into the conventional locations and for confirmation of the catheter positions. Once suitable His signals were acquired, the area was tagged on the mapping screen. The decision to use electroanatomical mapping (EAM) was operator‐dependent, as was the choice of EAM system. EAM systems available include EnSite Precision or X (St. Jude Medical, St Paul, MN, USA), CARTO 3D (Biosense Webster Inc., Diamond Bar, CA, USA), or Rhythmia (Boston Scientific, San Jose, CA, USA). The CardioLab EP recording system (GE Healthcare, Cleveland, OH, USA) was utilized for all electrophysiology studies. Standard protocols and procedures, depending on the arrhythmic substrate, were used for all ablation procedures.

Baseline clinical characteristics collected include patients' age, sex, comorbidities (structural heart disease and/or ischemic heart disease), type of SVT, and history of prior ablation. The type of SVT was confirmed by electrophysiological study. We also categorized patients into single right or left‐sided ablations based on the cardiac chamber location of the final RFCA lesion that terminated the clinical tachycardia reliably and rendered it non‐inducible. Patients with multiple pathways were categorized separately.

Procedural characteristics collected include the use of EAM systems, use of stereotaxis, use of ICE, procedure time (defined as the time interval from the initial access site puncture to the removal of all catheters), radiofrequency application time, fluoroscopy time, fluoroscopy dose area product (DAP), and total skin dose.

Outcomes collected include procedural success and complications. Procedural success was defined based on type of SVT: (i) AVNRT—the absence of inducible tachycardia for more than 20 min after the last RFCA application and no more than a single atrioventricular nodal echo with intravenous isoprenaline challenge; (ii) AVRT—non‐inducibility of tachycardia, loss of pre‐excitation (if manifested) and/or loss of retrograde accessory pathway conduction after 20 min of observation following the last RFCA application; (iii) AT—the inability to reinitiate the tachycardia despite intravenous isoprenaline challenge or programmed electrical stimulation down to atrial effective refractory period (ERP). Complications include those that occurred intra‐procedurally and during the immediate post‐procedure period.

Statistical analysis was performed using SPSS version 29.0 (SPSS Inc., Chicago, IL, USA). Continuous variables were tested for normality using the Kolmogorov–Smirnov test and presented as mean ± standard deviation. The Student *T*‐test and Mann–Whitney *U*‐test were used as appropriate. Categorical variables were analyzed using the Chi‐squared test when all expected cell counts were ≥ 5, and the Fisher's exact test was used when any expected cell count was < 5.

## 3 Results

**FIGURE 1:**
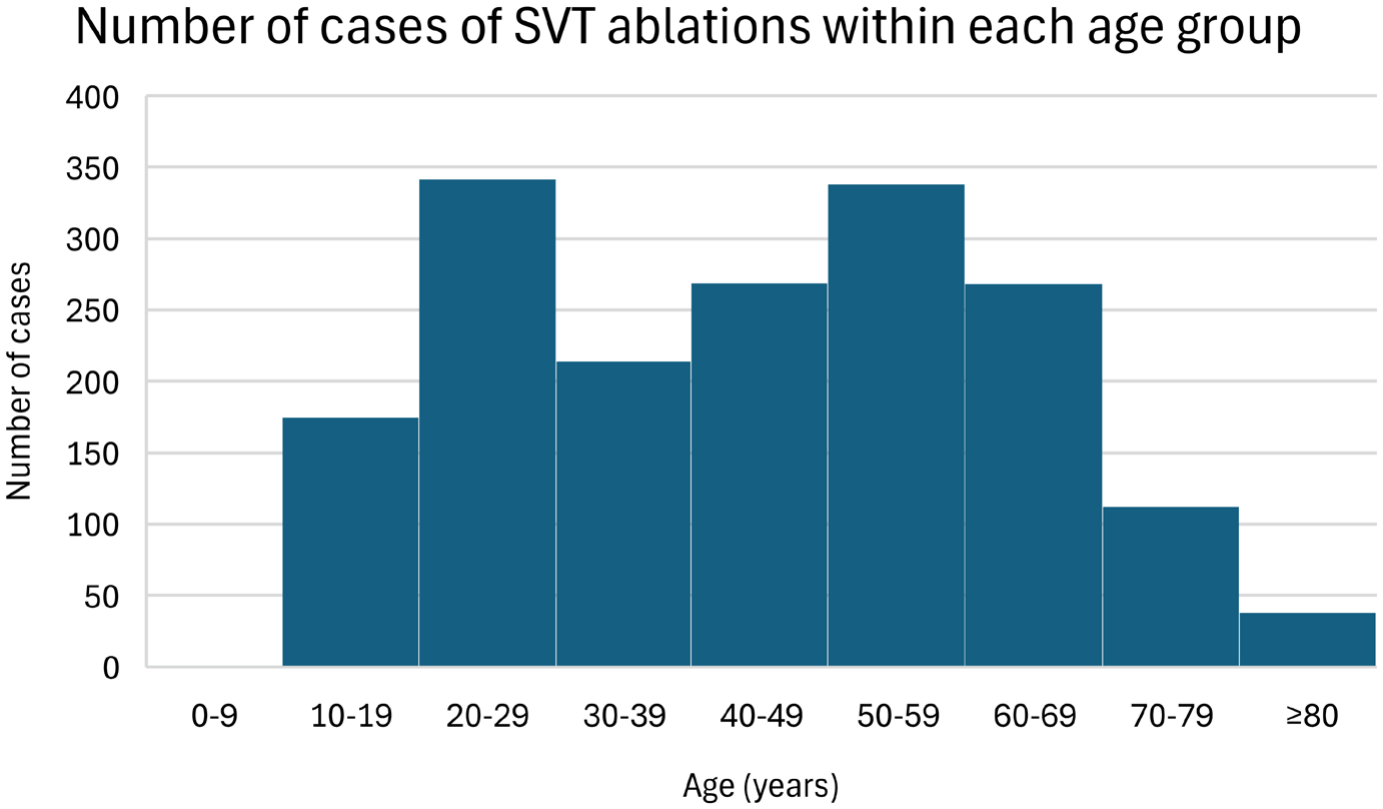
Histogram showing the number of cases of supraventricular tachycardia (SVT) ablations within each age group.

Over this 11‐year period, there were a total of 1758 cases of SVT ablation included in this study. One thousand six hundred and eight patients were aged < 70 years, 150 patients were aged ≥ 70 years. The youngest and oldest patients were aged 8 and 92 years old respectively. The mean age was 44.0 ± 18.9 years. The case distribution across the different age groups is illustrated in Figure 1. Approximately half of the patients were males (53.0%).

Patients aged ≥ 70 years were compared against patients who were aged < 70 years as summarized in Table 1. Between the two cohorts, there were no significant differences in sex, history of prior ablation, and the proportion of cases diagnosed with AT (*p* > 0.05). Elderly patients were more likely to have underlying structural heart disease and/or ischaemic heart disease (17.3% vs. 5.7%, *p* < 0.001). Elderly patients were also more likely to have a diagnosis of AVNRT (86.7% vs. 55.4%, *p* < 0.001) and less likely to have a diagnosis of AVRT (14% vs. 45.8%, *p* < 0.001).

**TABLE 1:** Comparison of clinical and procedural characteristics, and clinical outcomes between the patients < 70 and ≥ 70 years old during supraventricular tachycardia ablation.

	No. of missing cases (%)	Age < 70 (*n* = 1608)	Age ≥ 70 (*n* = 150)	*p*
Clinical characteristics				
Age—year	0 (0)	41 ± 16.7	76.4 ± 5.2	—
Female sex—no. (%)	0 (0)	752 (46.8)	74 (49.3)	0.547
Underlying structural heart disease and/or ischemic heart disease—no. (%)	**0 (0)**	**91 (5.7)**	**26 (17.3)**	**< 0.001**
Prior ablation—no. (%)	0 (0)	104 (6.5)	7 (4.7)	0.386
AVNRT—no. (%)	**0 (0)**	**891 (55.4)**	**130 (86.7)**	**< 0.001**
AVRT—no. (%)	**0 (0)**	**736 (45.8)**	**21 (14.0)**	**< 0.001**
AT no. (%)	0 (0)	41 (2.5)	6 (4.0)	0.292
Procedural characteristics				
Use of electro‐anatomical mapping—no. (%)	0 (0)	1085 (64.4)	110 (73.3)	0.141
Use of stereotaxis—no. (%)	0 (0)	30 (1.9)	1 (0.7)	0.512
Use of intracardiac echocardiography—no. (%)	0 (0)	76 (4.7)	7 (4.7)	0.974
Right‐sided SVT ablation—no. (%)	**0 (0)**	**1061 (66.0)**	**128 (85.3)**	**< 0.001**
Left‐sided SVT ablation—no. (%)	**0 (0)**	**443 (27.5)**	**13 (8.7)**	**< 0.001**
> 1 SVT ablated during procedure—no. (%)	0 (0)	93 (5.8)	8 (5.3)	0.821
Procedure time—minutes	**252**	**103.8**	**94.9**	**0.024**
	**−14.3**	**±48.1**	**±44.9**	
Radiofrequency application time—seconds	**343**	**387.6**	**326.2**	**0.032**
	**−19.5**	**±366.1**	**±299.3**	
Fluoroscopy time—minutes	**240**	**16.8**	**12.4**	**0.039**
	**−13.7**	**±20.6**	**±13.3**	
Fluoroscopy dose area product—mGy × cm^2^	250	13439.40	10123.00	0.087
	−14.2	±40048.3	±17724.4	
Total skin dose—mGy	**249**	**283.3**	**148**	**0.038**
	**−14.2**	**±3542.9**	**±599.7**	
Outcomes				
Complications—no. (%)	0 (0)	7 (0.4)	1 (0.7)	0.511
Successful ablation—no. (%)	0 (0)	1597 (99.3)	149 (99.3)	0.98

*Note:* Bold values indicate *p* < 0.05.

The use of EAM systems, stereotaxis, or ICE intra‐procedurally did not differ significantly between the cohorts (*p* > 0.05). Elderly patients were more likely to undergo right‐sided SVT ablation (85.3% vs. 66.0%, *p* < 0.001). The proportion of patients who had > 1 SVT ablated during the procedure did not differ significantly between the two cohorts (*p* > 0.05). Procedure time was significantly shorter in the elderly cohort than the younger cohort (94.9 ± 44.9 min vs. 103.8 ± 48.1 min, *p* = 0.024). Radiofrequency application time was significantly shorter in the elderly cohort than the younger cohort (326.2 ± 299.3 s vs. 387.6 ± 366.1 s, *p* = 0.032). The fluoroscopy time was significantly shorter in the elderly cohort (12.4 ± 13.3 min vs. 16.8 ± 20.6 min, *p* = 0.039) and total skin dose was significantly less in the elderly cohort (148.0 ± 599.7 mGy vs. 283.3 ± 3542.9 mGy, *p* = 0.038).

There were no significant differences in the number of complications (*p* > 0.05). In the younger cohort, out of the 1608 cases, there were 7 cases with complications. These include 2 cases of vascular access site haematomas that did not require intervention, 1 case of ventricular fibrillation during manipulation of the His catheter, and 4 cases of pericardial effusions of which 2 resulted in cardiac tamponade requiring pericardiocentesis. In the elderly cohort, out of the 150 cases, there was 1 case with a complication (cardiac tamponade requiring pericardiocentesis).

The acute procedural success rates were high in both cohorts at 99.3% and did not differ significantly.

## 4 Discussion

In this single‐centre study, we provide contemporary data on the clinical and procedural characteristics and outcomes of elderly patients (≥ 70 years) who undergo SVT. As anticipated in this study, the elderly were significantly more likely to have underlying structural heart disease and/or ischaemic heart disease (17.3% vs. 5.7%, *p* < 0.001). Previous studies have demonstrated that AVNRT and AT are more prevalent in elderly patients, and AVRT is more prevalent in younger patients [2]. These findings were mirrored in our group of patients who underwent SVT ablation; AVNRT was more common in the elderly cohort (86.7% vs. 55.4%, *p* < 0.001), and AVRT was less common in the elderly cohort (14.0% vs. 45.8%, *p* < 0.001). There was, however, no significant difference in the prevalence of AT between the two cohorts. Accordingly, elderly patients were significantly more likely to undergo right‐sided catheter SVT ablation (85.3% vs. 66.0%, *p* < 0.001) and less likely to undergo left sided SVT ablation (8.7% vs. 27.5%, *p* < 0.001). This is likely explained by the higher prevalence of AVNRT in the elderly cohort as well as higher prevalence of AVRT in the young cohort, of which 60%–70% are left sided in origin [9].

In addition, the prevalence of patients with AT who undergo RFCA in our study is seemingly low at 2.5% for the younger cohort and 4% only for the elderly cohort. This contrasts with previous studies, which report a higher prevalence of AT among patients undergoing SVT ablation, particularly in older patients, with prevalence rates in those over 70 years reported to be as high as 23% [10]. This may reflect geographic differences in patient selection or choice—that elderly Asian patients with AT may be less likely to be offered or elect to undergo catheter ablation in view of lower success rates or choose not to undergo an invasive procedure.

In our study, there were similar proportions of males and females in both the younger and elderly cohorts. This is despite the fact that SVT is more common in females due to sex‐specific differences in cardiac electrophysiology [11, 12]. This suggests that females too may be less likely to undergo catheter ablation, accounting for the similar proportions of males and females seen in our study, in keeping with what has been described in other studies [11, 12, 13].

Comparing the procedural characteristics between the two groups, the elderly cohort had a significantly shorter procedure time, radiofrequency application time, fluoroscopy time, and total skin dose (*p* < 0.05). This is likely related to the higher prevalence of AVNRT and right‐sided ablation in the elderly cohort that negates the need for trans‐septal puncture or retrograde aortic approach to gain access to the left side of the heart.

It is important to highlight that though the fluoroscopy time was significantly less in the elderly cohort (12.4 vs. 16.8 min), it was not insignificant. Thus, if SVT ablation were to be more widely offered to older patients, steps should be taken to minimize fluoroscopy use given the potentially harmful stochastic and deterministic effects of ionizing radiation on patients and staff [14]. These strategies include using the lowest exposure settings necessary and utilizing EAM systems. EAM systems have been consistently shown to reduce fluoroscopy use, with the most significant reduction seen in cases of AVNRT [15, 16], which is also the most common SVT in the elderly cohort. In our study, EAM systems were utilized 73.3% of the patients in the elderly cohort. Yet the fluoroscopy time was 12.4 min, higher than that reported in other studies on minimal fluoroscopy approaches to SVT ablation [15, 16, 17]. This may be explained by the fact that EAM systems were gradually adopted in our institution during the study period and there was a learning curve involved [18]. The presence of fellows in training is also known to increase fluoroscopy time [15]. Given the above, we would expect fluoroscopy use and fluoroscopy time to be lower for future cases of SVT ablation, and the use of EAM systems should be encouraged.

In our study, the complication rates were low (< 1%), similar to what has been reported in literature [19, 20] and did not differ significantly between the two cohorts. None of the complications were fatal or resulted in serious long‐term sequelae, though urgent treatment was required in some cases (e.g., urgent pericardiocentesis in the cases of cardiac tamponade and urgent defibrillation in the case of ventricular fibrillation). Left‐sided SVT ablation, which is less common in the elderly, would theoretically carry a higher risk of complications than right‐sided SVT ablation given the need for left‐sided access through either a trans‐septal or retrograde aortic approach and heparin use. The retrograde aortic approach is associated with increased risk of stroke, likely due to the embolization of debris from the arterial system [21], which would be of greater concern in the elderly as they are more likely to have atherosclerotic aortic disease. Thus, when approaching SVT ablation in elderly patients, it is essential to consider whether left‐sided access is likely to be required, and if so a trans‐septal approach may be preferred over a retrograde aortic approach. One of the biggest concerns with AVNRT ablation in the elderly is the theoretical risk of iatrogenic complete heart block (CHB). In our elderly cohort, there were no cases of post‐ablation CHB despite an 86.7% prevalence of AVNRT. Reported long term outcome of AVNRT ablation in patients ≥ 75 years were also excellent with no occurrence of CHB with an average follow‐up of 37 months as well [22]. The acute success rates in both cohorts were high (99.3%), comparable to previous studies [22, 23, 24, 25].

Overall, the data suggests that despite differences in clinical characteristics between older and younger patients undergoing SVT ablation, SVT ablation appears to be a safe and effective treatment in both the young and old. The favorable results seen in the elderly cohort may also be attributed to the fact that there was a higher proportion of AVNRT and right‐sided ablations. In an increasingly aging population with advancements in healthcare, the number of elderly patients presenting with SVT and seeking treatment is likely to increase. Appropriate medical treatment should not be restricted by virtue of age or comorbidities alone given good safety and efficacy outcomes. Further tools for risk stratification such as frailty indices may further assist in elderly patient selection for SVT ablation while minimizing complications.

Several limitations exist, in particular the lack of follow‐up data, with no information on long‐term complication rate and clinical outcomes. Whilst the majority of the complications occur on the day of the procedure, some may develop later and be associated with considerable morbidity [20]. For instance, AVNRT ablation is associated with a small but increased risk of late‐onset atrioventricular block and pacemaker implantation that can occur several years after ablation. However, the risk of late pacemaker implantation is low (< 1%) [26, 27]. There are previous studies that suggest that even after taking into account the late complications and long‐term sequelae, the overall complication rate is low and catheter ablation remains a safe option in older patients [28, 29]. Our data only includes patients who underwent SVT ablation, and did not include all patients who have a diagnosis with SVT. Thus, the positive results seen across the board, including those in the older cohort when compared to the younger cohort, may be a result of selection bias. Data on hard outcomes such as hospitalization rates and patient‐reported quality of life are lacking. Hence, it is assumed that the high acute procedural success rates seen in our cohort translates to good long‐term patient outcomes. Supporting this assumption, previous studies have suggested that acutely successful SVT ablation is associated with favorable long‐term outcomes and significant improvements in quality of life [24, 25, 30, 31].

Frailty, though common in elderly patients, was not assessed in our cohort. While frailty is strongly correlated with age [32], it should ideally be formally assessed in each patient. Although no studies to date have specifically examined the impact of frailty on SVT ablation outcomes, existing literature has shown that frailty is associated with worse outcomes following AF ablation in patients with HFrEF and after percutaneous coronary intervention [33, 34]. Thus, it is plausible that frailty may also negatively impact SVT ablation outcomes and should be considered during patient selection. In addition, data on patient autonomy and other comorbidities were not available in this study, but are also important considerations and should be part of the clinical decision‐making process.

## 5 Conclusions

Data from our study suggests that catheter ablation for SVT is a safe and effective treatment and should be considered in all symptomatic patients including elderly patients. The type of SVT and presence of comorbidities, including frailty, are likely important factors to consider during the clinical decision‐making process. Further studies examining these factors, along with long‐term outcomes, may help refine patient selection and optimize treatment strategies.

## Funding

The authors have nothing to report.

## Ethics Statement

Ethical review and approval were waived for this study due to use of anonymized data from another registry.

## Consent

The authors have nothing to report.

## Conflicts of Interest

The authors declare no conflicts of interest.

## Data Availability

Data is unavailable due to privacy or ethical restrictions.
